# Exploring the moderating role of health-promoting behaviours and self-compassion on the relationship between clinical decision-making and nurses’ well-being

**DOI:** 10.1177/17449871241270822

**Published:** 2024-09-28

**Authors:** Molly Miley, Michail Mantzios, Helen Egan, Kathrina Connabeer

**Affiliations:** PhD student, Department of Psychology, Birmingham City University, UK; Professor of Applied and Experimental Psychology, Department of Psychology, Birmingham City University, UK; Professor of Health Psychology, Department of Psychology, Birmingham City University, UK; Senior Lecturer in Psychology, Department of Psychology, Birmingham City University, UK

**Keywords:** diet and eating, health promotion, mental health, nurses, nursing practice

## Abstract

**Background:**

Clinical decision-making is an essential part of the nursing role and has implications for both patient care and nurses’ well-being.

**Aim:**

This study aimed to explore the relationship between nurses’ perceptions of clinical decision-making ability and moral distress across a nursing population, and the potential link to self-compassion and health-promoting behaviours.

**Methods:**

A self-report questionnaire was distributed to a sample of nurses (*N* = 152) from April to September 2022. The survey explored nurses’ perceptions of clinical decision-making ability, moral distress, physical activity, grazing, stress-eating, burnout and self-compassion.

**Results:**

Perceived clinical decision-making ability was associated with moral distress experience, and both self-compassion and grazing moderated this relationship, independently.

**Conclusion:**

Findings highlight the link between nurses’ perceptions of clinical decision-making ability and moral distress experience. Both eating behaviours and self-compassion influence the relationship between these two factors and identify potential areas that may support (and hinder) nurses’ well-being through clinical decision-making. These findings reinforce the importance of healthy eating habits and being self-compassionate to prevent moral distress arising as a result of clinical decision-making.

## Introduction

Decision-making remains a pivotal part of nursing, requiring individuals to make decisions on patient diagnosis, intervention and interactions (Smith et al., 2008). Such decisions are guided by personal values, experience and most importantly professional knowledge (Smith et al., 2008). However, continuous developments across scientific knowledge and technology over recent years have accelerated decision-making responsibilities, with nurses having to make additional decisions regarding resuscitation and termination of life-support (Numminen and Leino, 2007). Nurses are, therefore, required to consider additional factors in the decision-process, whilst being constrained by external sources, such as resource availability and staffing levels (Berhie et al., 2020; Bucknall, 2003). Such constraints can prevent nurses from aligning their actions with the principles taught during their training, resulting in ethical conflicts (Park et al., 2003). When left unresolved, ethical conflicts can cause moral distress, leaving nurses increasingly vulnerable to a wide range of well-being issues (Corley et al., 2005; Smallwood et al., 2021).

Moral distress is a common phenomenon among healthcare workers (Almutairi et al., 2019), one that often stems from ongoing ethical problems and conflicts (Humphries and Woods, 2016; Woods, 2020). This psychological response occurs when one feels constrained from acting on what they believe to be morally correct (British Medical Association, 2021). Such violation of one’s moral values or duties can not only compromise patient care but also prove detrimental to individual well-being (McAndrew et al., 2018; Smallwood et al., 2021). Specifically, literature has identified moral distress as a key contributor and root cause of burnout amongst clinicians (Dzeng and Curtis, 2018; Rushton et al., 2015). Burnout is defined as the state of physical or emotional exhaustion stemming from chronic, unresolved or occupation-related stress (World Health Organization, 2019) and relates to higher patient infection, greater patient dissatisfaction and a higher incidence of medication errors amongst healthcare professionals (Hall et al., 2016; Van Bogaert et al., 2014). Further associations have been drawn with decision-making specifically, with burnout predicting more avoidant and irrational decision-making styles (Michailidis and Banks, 2016). Therefore, if nurses’ decision-making and well-being are to be supported, it is important to identify modifiable areas to minimise burnout and moral distress experiences.

One possible strategy is increasing nurses’ levels of physical activity. Physical activity describes any bodily movement produced by skeletal muscles that results in energy expenditure (Caspersen et al., 1985) and is linked to reduced burnout, lower emotional stress and greater psychological well-being (Cooper and Barton, 2016; Naczenski et al., 2017). Further associations have been drawn between physical activity and resilience, with individual competence and autonomy mediating this relationship (Xu et al., 2021). However, evidence suggests that a large proportion of nurses do not undertake sufficient physical activity to reap the full benefits of exercise (Kyle, 2022; Malik et al., 2011). Thus, physical activity is something to consider when seeking to promote nurses’ health and well-being outcomes.

Alongside physical activity, eating behaviours have been identified as an important health behaviour, relating to higher levels of self-efficacy and lower levels of psychological distress (Głąbska et al., 2020). Healthy eating practices are particularly important for buffering the impact of stressors on well-being and have been linked to lower levels of burnout, depression, anxiety and post-traumatic stress disorder, independently (Alexandrova-Karamanova et al., 2016; Hall et al., 2015; Luong et al., 2021). However, evidence suggests that nurses tend to turn to unhealthy eating behaviours to cope with feelings of stress and accommodate the shift-work nature of the role (Almajwal, 2016). Notably, higher levels of stress are associated with increased consumption of ultra-processed and hyperpalatable food (Cortes et al., 2021; Yau and Potenza, 2013), whilst irregular work schedules and inadequate workplace facilities have been seen to encourage nurses to skip meals (Almajwal, 2016; Gupta et al., 2019; Nicholls et al., 2017). Skipping meals can lead to greater grazing tendencies (Northwell Health, 2020).

Grazing is defined as the uncontrolled and repetitive eating of small amounts of food (Lane and Szabó, 2013) and is associated with eating disorders, psychological distress and reduced quality of life (Colles et al., 2008; Spirou et al., 2023). Research on grazing is limited, particularly in nursing professions, and has not yet been explored in relation to occupational stresses such as moral distress and clinical decision-making. Hence, it is important to consider the role of grazing and eating behaviours within the moral distress and clinical decision-making context, with further consideration to elements that can promote healthier eating practice.

An area implicated in the uptake of healthier lifestyle decisions, particularly regarding physical activity and eating practices is self-compassion (Hussain et al., 2022; Mantzios et al., 2018a; Phillips & Hine, 2021). Self-compassion can be defined as being understanding towards the self during times of suffering and is centred around three core elements: self-kindness, common-humanity and mindfulness (Neff, 2003). Recent findings suggest that self-compassion is not only negatively related to grazing (Mantzios et al., 2018b) but also predicts greater physical health and health behaviour (Egan et al., 2019; Phillips and Hine, 2021). It has also been found to positively predict daily eating behaviour through the reduction of perceived stress (Li et al., 2020). Given these positive associations, it is unsurprising that self-compassion has been repeatedly linked to greater well-being amongst nursing professionals, predicting lower levels of burnout and reduced mental health problems (Abdollahi et al., 2021; Kotera et al., 2021). Therefore, the present study seeks to examine the role of self-compassion and health-promoting behaviours in relation to clinical decision-making and nurses’ well-being, with the goal of supporting nurses through the decision-making process.

## Methods

### Design

This study utilised a cross-sectional design. Data were collected from April to September 2022.

### Participants

Volunteer and snowball sampling was used to recruit 152 nurses from across the United Kingdom (*M*^age^ = 42, SD = 9.7, *M*^BMI^ = 29.35, SD = 7.96). Eligibility criteria included individuals who were over the age of 18, were currently practising within the United Kingdom and had worked in the nursing profession for at least 6 months. This criterion ensured that participants possessed sufficient knowledge of the clinical decision-making process to meet the research aims (Cowin and Hengstberger-Sims, 2006). Cohen’s (1992) guidelines suggest that to achieve a medium effect size, with alpha set at 0.01 and a power of 0.80, a minimum of 147 participants were required to conduct a regression analysis. See Table 1 for summary of participant characteristics.

**Table 1.** Participant characteristics.

Characteristic	*n*	%
Gender		
Female	134	88.2
Male	18	11.8
Do you smoke?		
Yes	15	9.9
No	137	90.1
Ethnicity		
White-British	136	89.5
Irish	6	3.9
Other	10	6.6
Speciality		
Adult health	33	21.7
Psychiatric/mental health	20	13.2
Community	15	9.9
General medicine/surgery	9	5.9
Critical care	17	11.2
Oncology	9	5.9
Parent/child health	9	5.9
Other	112	26.3
Descriptive statistics for continuous variables		
	*M*	SD
Age	42.41	9.7
BMI	29.35	7.96
Years spent in profession	17.68	11.59
Hours practised per week	36.91	7.42
Weekly alcohol consumption	6.02	6.95

### Measurements

Demographic questionnaire: Participants were asked to report their age, gender, ethnicity, years spent in the nursing profession, hours worked each week, clinical speciality, banding position, height, weight, smoking behaviours and weekly alcohol consumption.

The Sussex-Oxford Compassion for the Self-Scale (SOCS; Gu et al., 2019). The SOCS consists of 20 items, measuring compassion directed towards oneself. The items were arranged into five subscales: recognising suffering, understanding the universality of suffering, feeling for the person suffering, tolerating uncomfortable feelings, acting or being motivated to act to alleviate suffering. Sample items include ‘I’m good at recognising when I’m feeling distressed’. Responses ranged from 1 (not true at all) to 5 (always true), with a higher score indicating greater levels of self-compassion. Cronbach’s alpha for the present study was α = 0.937 for total score, α = 0.864 for recognising suffering, α = 0.763 for understanding the universality of suffering, α = 0.874 for feeling for the person suffering, α = 0.798 for tolerating uncomfortable feelings and α = 0.881 for acting or being motivated to act to alleviate suffering.

The Moral Distress-Scale-Revised (MDS-R; Hamric et al., 2012). The MDS-R consists of 21 items, designed to assess experiences of moral distress. Participants are required to respond to statements in terms of frequency, and intensity, independently. Example items include, ‘Be required to care for patients I don’t feel qualified to care for’. Responses range from 1 (Never/None) to 5 (Very frequently/A great extent), with a higher score indicating greater levels of moral distress. Cronbach’s alpha for the present study was α = 0.933.

The Clinical Decision-Making in Nursing Scale-40 (CDMNS-40; Jenkins, 1983). The CDMNS-40 consists of 40 items designed to measure perceptions of clinical decision-making ability across the nursing profession. The items are arranged into four subscales: Search for alternatives or options, canvassing of objectives and values, evaluating and re-evaluation of consequences and search for information and unbiased assimilation of new information. Sample items include ‘I consider even the remotest consequences before making a choice’. Responses range from 1 (never) to 5 (always), with a higher score demonstrating a greater perception of clinical decision-making ability. Cronbach’s alpha for the present study was α = 0.710 for the total score, α = 0.134 for search for alternatives or options, α = 0.495 for canvassing of objectives and values, α = 0.598 for evaluating and re-evaluation of consequences and α = 0.255 for search for information and unbiased assimilation of new information. Reliability scores for each of the four subscales fall below an acceptable level of reliability and have not been used in the analysis (Tavsancil, 2006).

The Clinical Decision-Making in Nursing Scale-13 (CDMNS-13; Miley et al., 2023) The CDMNS-13 is a revised shorter version of the clinical decision-making in nursing scale (Jenkins, 1983) and consists of 13 items designed to measure perceptions of decision-making ability across the nursing profession. The scale is unidimensional and reflects mixed findings in past literature, and an inability for subscales to display adequate internal consistency (see Miley et al., 2023). The scale includes items such as ‘Looking for new information in making a decision is more trouble than it’s worth’. Responses range from 1 (never) to 5 (always), with a higher score indicating a greater perception of clinical decision-making ability. Cronbach’s alpha for the present study was α = 0.710. The CDMNS-13 is reported in parallel to the CDMNS-40 and will be presented in parentheses throughout the results section.

The Oldenburg Burnout Inventory (OBI; Demerouti et al., 2002). The OBI consists of 16 items, designed to assess experiences of burnout. The items were arranged into two subscales: Disengagement and Exhaustion. Sample items include ‘I always find new and interesting aspects in my work’. Responses ranged from 1 (strongly agree) to 4 (strongly disagree), with a higher score indicating greater levels of burnout. Cronbach’s alpha for the present study was α = 0.904 for total score, α = 831 for disengagement and α = 0.861 for exhaustion.

Salzburg Stress-Eating Scale (SSES; Meule et al., 2018). The SSES consists of 10 items, designed to assess stress-eating tendencies. Sample items include ‘When I am overwhelmed with things I have to do’. Responses range from 1 (I eat much less than usual) to 5 (I eat much more than usual), with a higher score indicating greater engagement with eating when stressed. Cronbach’s alpha for the present study was α = 0.931.

The Grazing Questionnaire (GQ; Lane and Szabó, 2013). The GQ consists of eight items, designed to assess self-reported engagement with grazing eating behaviours. Participants are asked to rate themselves against statements such as ‘Do you find yourself taking extra helpings or picking at extra food once you’ve finished your main meal?’ Responses ranged from 0 (Never) to 4 (All of the time), with a higher total score indicating greater engagement with grazing eating behaviours. Cronbach’s alpha for the present study was α = 0.905.

The International Physical Activity Questionnaire-Short-Form (IPAQ-SF; International Consensus Group, 1988, as cited by Craig et al., 2003). The IPAQ-SF consists of seven questions, designed to assess engagement with physical activity. It covers five different activity domains, namely, physical activity related to work, transportation, housework, leisure-time activities and time spent sitting. Sample questions include, ‘How much time did you spend doing vigorous physical activities like heavy lifting, digging, aerobics or fast bicycling?’ The IPAQ-SF questions were used to estimate the amount of time individuals spend engaging with physical activity each week and were measured in metabolic equivalent of task minutes per week (MET-min-week). MET-min-week refers to the amount of energy expended whilst performing various activities per week (Jetté et al., 1990) and is used to measure engagement with walking, moderate activities and vigorous activities in the current study.

### Data collection

An online questionnaire survey was conducted from April to September 2022. Potential participants were introduced to the current study through social media platforms (Facebook, Twitter) and directed to an online platform (Qualtrics) to complete the survey.

### Ethical consideration

Ethical approval was obtained from Birmingham City University’s Ethics Committee in line with the Declaration of Helsinki (World Medical Association, 2013). Consent was obtained via an online form administered on the Qualtrics software.

### Data analysis

Descriptive statistics including means, standard deviations and frequencies were obtained to describe the characteristics of the sample. Pearson’s bivariate correlations were conducted to explore initial relationships between perceived clinical decision-making ability, moral distress, self-compassion, burnout and health behaviours. Moderation effects were determined using Hayes’ (2017) PROCESS macro (model 1), with a bootstrap sample of 5000. All analyses utilised IBM SPSS version 28.0 (the Statistical Package for Social Sciences) and PROCESS (Preacher and Hayes, 2008), with *p* values ⩽0.05 being accepted as statistically significant.

## Results

### Correlations

Bivariate correlations revealed that perceived clinical decision-making ability was significantly associated with moral distress experience across the nursing profession (*r* = −0.225, *p* = 0.005; CDMNS-13: *r* = −0.218, *p* = 0.007). Regarding health behaviours, perceived clinical decision-making ability also demonstrated negative associations with both stress-eating (*r* = −0.198, *p* = 0.015; CDMNS-13: *r* = −0.198, *p* = 0.014) and grazing (*r* = −0.207, *p* = 0.010; CDMNS-13: *r* = −0.194, *p* = 0.016), independently. This suggests that perceptions of clinical decision-making ability decrease as engagement with stress-eating and grazing behaviours increase. Further associations were drawn with physical activity; higher levels of moderate physical activity were associated with greater scores on the clinical decision-making scale (CDMNS-13: *r =* 0.176, *p* = .03), whereas neither walking nor vigorous activities were significantly associated.

Further correlation analyses into moral distress experience revealed significant associations with burnout (*r* = 0.532, *p* < 0.001) and self-compassion (*r* = −0.341, *p* = 0.001). It was the tolerating uncomfortable feelings dimension of self-compassion that demonstrated the strongest negative relationship with moral distress experience (*r* = -0.352, *p* < 0.001). Moreover, a significant relationship was also observed between moral distress and eating behaviours. Higher scores on the MDS-R were associated with greater stress-eating and grazing behaviours (see Table 2).

**Table 2.** Bivariate correlations of the relationships between all variables.

Variables	1	2	3	4	5	6	7	8	9	10	11	12	13	14	15	16	17	18
(1) CDMNS-13																		
(2) CDMNS-40	0.849**																	
(3) MDS-R	−0.218**	−0.225**																
(4) OBI	−0.065	−0.155	0.532**															
(5) OBI – D	−0.117	−0.214**	0.489**	0.922**														
(6) OBI – E	−0.003	−0.073	0.494**	0.924**	0.704**													
(7) SOCS	0.001	0.087	−0.341**	−0.572**	−0.435**	−0.620**												
(8) SOCS– RS	0.053	.066	−.253**	−.364**	−.242**	−.429**	.807**											
(9) SOCS−UUS	0.241**	0.256**	−0.197*	−0.232**	−0.166*	−0.262**	0.518**	0.455**										
(10) SOCS– FPS	−0.046	0.064	−0.310**	−0.560**	−0.448**	−0.585**	0.924**	0.653**	0.315**									
(11) SOCS– TUF	−0.019	0.054	−0.352**	−0.584**	−0.463**	−0.616**	0.887**	0.612**	0.300**	0.831**								
(12) SOCS– MTA	−0.128	−0.011	−0.259**	−0.518**	−0.390**	−0.565**	0.866**	0.543**	0.291**	0.820**	0.742**							
(13) SSES	−0.198*	−0.198*	0.169*	0.151	0.145	0.135	−0.009	−0.121	0.051	−0.014	0.019	0.045						
(14) GQ	−0.194*	−0.207*	0.281**	0.310**	0.244**	0.327**	−0.251**	−0.189*	−0.108	−0.218**	−0.265**	−0.216**	0.511**					
(15) IPAQ – W	0.053	0.106	−0.023	−0.094	−0.064	−0.110	0.142	0.058	0.020	0.147	0.145	0.175*	0.123	−0.107				
(16) IPAQ – M	0.180*	0.099	−0.134	−0.153	−0.141	−0.142	0.220**	0.216**	0.075	0.203*	0.207*	0.166*	−0.003	−0.144	0.248**			
(17) IPAQ – V	0.041	0.045	−0.095	−0.069	−0.025	−0.103	0.053	0.035	0.010	0.028	0.078	0.055	0.074	−0.111	0.287**	0.358**		
(18) IPAQ – T	0.110	0.111	−0.109	−0.139	−0.095	−0.158	0.177*	0.125	0.043	0.160*	0.187*	0.175*	0.097	−0.162*	0.725**	0.671**	0.783^**^	

CDMNS-13: 13-item Clinical Decision-Making in Nursing Scale; CDMNS-40: 40-item Clinical Decision-Making in Nursing Scale; MDS-R: Moral-Distress Scale-Revised; OBI: Oldenburg Burnout Inventory total; OBI-D: Oldenburg Burnout Inventory-Disengagement; OBI-E: Oldenburg Burnout Inventory-Exhaustion; SOCS: Sussex-Oxford Compassion for Self-Scale total; SOCS-RS: Sussex-Oxford Compassion for the Self-Scale-Recognising Suffering; SOCS-UUS: Sussex-Oxford Compassion for the Self-Scale-Understanding the Universality of Suffering; SOCS – FPS: Sussex-Oxford Compassion for the Self-Scale – Feeling for the Person Suffering; SOCS-TUF: Sussex-Oxford Compassion for the Self-Scale- Tolerating Uncomfortable Feelings; SOCS-MTA: Sussex-Oxford Compassion for the Self-Scale – Motivated to Act to Alleviate suffering; SSES: Salzburg Stress-Eating Scale; GQ: Grazing Questionnaire; IPAQ-W; Walking METS per week on the International Physical Activity Questionnaire; IPAQ-M: Moderate Physical Activity METS per week on the International Physical Activity Questionnaire; IPAQ – V – Vigorous METS per week on the International Physical Activity Questionnaire; IPAQ–T: Total METS per week for physical activity.

* Is statistically significant at *p* < 0.05.

** Is statistically significant at *p* < 0.001.

### Moderations

The first moderation model used CDMNS-13 as the predictor, moral distress as the dependant and grazing as a moderator. Grazing behaviours significantly moderated the relationship between perceived clinical decision-making ability and moral distress (*F*(3, 147) = 6.14, *p* < 0.001, *r*^2^ = 0.111). Simple slope analyses revealed that average and high levels of grazing weakened the relationship between these variables, suggesting that the negative relationship between nurses’ perceptions of clinical decision-making ability and moral distress becomes significant as grazing scores increase (see Table 3).

**Table 3.** Conditional effects of the subscales of self-compassion and grazing on the relationship between CDMNS-13 and moral distress (*N* = 151).

Predictors	Moderator value	*β*	*p*	95% CI	
				Lower	Upper
Tolerating uncomfortable feeling (SOCS)	−1 SD	−4.37	**<0.001**	−6.85	−1.88
	At the mean	−2.68	**0.004**	−4.47	−0.892
	+1 SD	−.993	0.416	−3.40	1.41
Grazing	−1 SD	−.110	0.929	−2.55	2.33
	At the mean	−2.13	**0.027**	−4.01	−0.251
	+1 SD	−4.15	**0.001**	−6.58	−1.72

SD: standard deviation; CI: confidence intervals; p: significance level; β: regression coefficient.

Bold indicates significance.

A second model used CDMNS-13 as the predictor, moral distress as the dependant and the tolerating uncomfortable feelings dimension of self-compassion as a moderator. Results revealed that the tolerating uncomfortable feelings subscale significantly shifted the relationship between perceived clinical decision-making ability and moral distress, being a significant moderator (*F*(3, 147) = 9.99, *p* < 0.001, *r*^2^ = 0.169). Simple slope analyses revealed that average and low levels of tolerating uncomfortable feelings significantly weakened the relationship between nurses’ perceptions of clinical decision-making ability and moral distress, suggesting that the relationship only becomes significant when self-compassion scores decrease (see Table 3).

## Discussion

The present study aimed to investigate the impact of clinical decision-making on nurses’ well-being. Initial analyses revealed that as perceived clinical decision-making ability increases, reports of moral distress experience decrease across the nursing sample. This aligns closely with existing literature on decision-making, where adaptive decision-making strategies and decision-making competency have been seen to positively influence health and well-being outcomes (Páez-Gallego et al., 2020; Ravneet and Kawaljit, 2021). The present study builds upon these findings in a clinical environment and extends its implications to moral distress experience directly.

In response to the observed relationship between perceived clinical decision-making ability and moral distress, the present study sought to investigate potential areas that may influence the strength of these associations. Results revealed that both stress-eating and grazing were significantly associated with both perceived clinical decision-making ability and moral distress, independently. However, further analyses revealed that it was only grazing behaviours that moderated the relationship between perceived clinical decision-making ability and moral distress experience, suggesting that as grazing behaviours increased, the negative relationship between clinical decision-making and moral distress was strengthened. Although there is limited research on the effect of grazing on well-being across the nursing demographic, Heriseanu et al. (2019) found that frequency of grazing was associated with lower mental health-related quality of life. Grazing categorised as being compulsive has also been associated with a wealth of negative health outcomes, including anxiety, depression and eating disorders (Heriseanu et al., 2019; Spirou et al., 2023). This alignment of previous research to present findings suggests that grazing behaviours should be considered when designing an intervention to support nurses’ well-being whilst navigating the everyday decision-making and moral aspects of their work. Perhaps integrating regular breaks would allow sufficient time for more regulated eating behaviours and reduce the role of clinical decision-making in nurses’ experience of moral distress.

Self-compassion was explored for its potential when influencing the relationship between perceived clinical decision-making ability and moral distress. Existing research emphasises the positive influence of self-compassion on well-being, stress and life satisfaction (Li et al., 2021; Stutts et al., 2018). Past research is consistent with the findings of the present study, where higher levels of self-compassion were associated with lower levels of both moral distress and burnout, independently. Further analyses revealed that it was only the *tolerating uncomfortable feelings* dimension of self-compassion that moderated the relationship between perceived clinical decision-making ability and moral distress. Notably, as scores on tolerating uncomfortable feelings increased, the negative relationship between clinical decision-making and moral distress was weakened. This suggests that being self-compassionate may be protective against the impact of clinical decision-making on nurse’s experience of moral distress. These findings lend insight into the multidimensional nature of self-compassion, recognising that enhancing certain elements may be more effective than others in supporting nurses through the decision-making process. This knowledge should be integrated into potential support strategies when seeking to promote nurses’ well-being.

Consideration needs to be given to the differentiation of findings between the CDMNS-40 and CDMNS-13, the latter developed due to inadequate internal consistency for CDMNS-40 subscales and potential limitations imposed by its length on clinical decision-making inquiries. This shorter psychometric scale can enhance efficiency, diminish respondent fatigue, improve retention and reduce administration costs while increasing accessibility. In the current research, the shorter version demonstrated successful moderations of relationships, emphasising its validity and equivalence to the CDMNS-40. Both the *tolerating uncomfortable feelings* dimension of self-compassion and grazing significantly influenced the relationship between CDMNS-13 and moral distress, with greater perceptions of clinical decision-making ability predicting lower levels of moral distress through self-compassion, and higher levels of moral distress through grazing. However, the CDMNS-40 did not demonstrate any significant moderations. Future studies should further explore the utility of the CDMNS-13, both independently and in conjunction with the CDMNS-40, to fully validate its effectiveness across known risk indicators in health professions.

### Limitations

The present study has two limitations to acknowledge. Firstly, participants of the present study predominantly identified as White-British (89.5%), resulting in ethnic under-representation, when compared to national statistics (Baker, 2022). With Range and Rotherham (2010) finding ethnicity to influence moral distress experience, it is important that this study is replicated on a more diverse population to strengthen findings. Secondly, the cross-sectional nature of this study makes it difficult to infer cause and effect. To draw stronger conclusions, further research should utilise an experimental design to allow for more causal inferences to be made about the role of health behaviours and self-compassion on clinical decision-making and moral distress.

### Conclusion

In summary, these data contribute to existing knowledge on the impact of clinical decision-making across the nursing profession; both health behaviours and self-compassion demonstrate significant associations with perceived clinical decision-making ability and moral distress, which should be considered in potential intervention strategies. Given the interaction between grazing and moral distress, we highlight the importance of systemic support, in terms of break scheduling and meal opportunities. Supporting nurses in establishing healthier eating habits, and reducing grazing behaviours, may offer promising potential in the mitigation of moral distress. Additionally, the role of self-compassion in predicting reduced moral distress experience may be another element considered for potential intervention and support. If nurses are equipped with the skills and resources to practise self-compassion, they will be better protected from the impact of clinical decision-making. Existing research into the area suggests that self-compassion training and education workshops are effective at enhancing self-compassion across nursing professions (Franco and Christie, 2021). The present study therefore highlights the importance of systemic support and education opportunities when increasing self-compassion and the impact that this may have on nurse’s experience of moral distress. With moral distress being deemed an inherent part of the nursing role, strategies like these, which are more individual-focused, may offer long-term relief from the demands of nursing. We conclude that both self-compassion and health behaviours should be considered in the mitigation of moral distress if nurse’s well-being is to be supported.

**Key points for policy, practice and/or research**

- Nurses’ perceptions of clinical decision-making ability are associated with moral distress experience.
- Grazing behaviours negatively influenced the relationship between perceived clinical decision-making ability and moral distress across the nursing sample. Healthcare organisations should support scheduled breaks for meals to prevent grazing, and support nurses’ well-being.
- Self-compassion had a positive influence on the relationship between perceived clinical decision-making ability and moral distress. Elements of self-compassion should be considered when supporting nurses through the decision-making process.
